# Diagnostic Activities and Diagnostic Practices in Medical Education and Teacher Education: An Interdisciplinary Comparison

**DOI:** 10.3389/fpsyg.2020.562665

**Published:** 2020-10-20

**Authors:** Elisabeth Bauer, Frank Fischer, Jan Kiesewetter, David Williamson Shaffer, Martin R. Fischer, Jan M. Zottmann, Michael Sailer

**Affiliations:** ^1^Education and Educational Psychology, Department Psychology, LMU University of Munich, Munich, Germany; ^2^Institute for Medical Education, University Hospital, LMU University of Munich, Munich, Germany; ^3^Epistemic Analytics Lab, Department of Educational Psychology, University of Wisconsin Madison, Madison, WI, United States

**Keywords:** diagnostic activities, diagnostic practices, medical education, teacher education, interdisciplinary research

## Abstract

In this article, we investigate diagnostic activities and diagnostic practices in medical education and teacher education. Previous studies have tended to focus on comparing knowledge between disciplines, but such an approach is complicated due to the content specificity of knowledge. We compared 142 learners from medical education and 122 learners from teacher education who were asked to (a) diagnose eight simulated cases from their respective discipline in a simulation-based learning environment and (b) write a justificatory report for each simulated case. We coded all justificatory reports regarding four diagnostic activities: *generating hypotheses*, *generating evidence*, *evaluating evidence*, and *drawing conclusions.* Moreover, using the method of Epistemic Network Analysis, we operationalized diagnostic practices as the relative frequencies of co-occurring diagnostic activities. We found significant differences between learners from medical education and teacher education with respect to both their diagnostic activities and diagnostic practices. Learners from medical education put relatively more emphasis on generating hypotheses and drawing conclusions, therefore applying a more hypothesis-driven approach. By contrast, learners in teacher education had a stronger focus on generating and evaluating evidence, indicating a more data-driven approach. The results may be explained by different epistemic ideals and standards taught in higher education. Further research on the issue of epistemic ideals and standards in diagnosing is needed. Moreover, we recommend that educators think beyond individuals’ knowledge and implement measures to systematically teach and increase the awareness of disciplinary standards.

## Introduction

Interdisciplinary research involves various challenges, for example, the comparability of specific variables. In this article, we refer to a framework of diagnostic activities (Fischer et al., 2014; Heitzmann et al., 2019) that was applied to compare learners’ diagnostic assessments within two disciplines (i.e., medical education and teacher education). We aim to investigate diagnostic activities in these disciplines and explore their conceptual integration into diagnostic practices. Hereby, we also seek to facilitate future interdisciplinary research on diagnostic practices and the learning of diagnostic activities.

Facilitating diagnostic skills in higher education is an important objective in many disciplines (e.g., Chernikova et al., 2020). This is certainly the case in medical education, which focuses on training future physicians in the assessment of patient symptomology. Similarly, future teachers’ professional challenges include diagnosing students’ performance, progress, learning difficulties such as behavioral and learning disorders, or other learning prerequisites (Reinke et al., 2011). Independent of the discipline, we broadly define diagnosing as “a process of goal-oriented collection and integration of case-specific information to reduce uncertainty in order to make medical or educational decisions” (Heitzmann et al., 2019, p.4).

Professional knowledge is a crucial prerequisite for diagnosing (Blömeke et al., 2015). There are numerous models conceptualizing professional knowledge (e.g., Shulman, 1987; Kopp et al., 2009; Charlin et al., 2012), e.g., in terms of content like biological knowledge in medicine (Charlin et al., 2012) and pedagogical knowledge in teaching (Shulman, 1987). Research has even suggested that professional knowledge in diagnostic reasoning may not only be discipline-specific but case-specific, since abstract types of e.g., strategic knowledge (Kopp et al., 2009) do not seem to transfer well across cases (e.g., Wimmers et al., 2007; Schwartz and Elstein, 2009). A recently proposed interdisciplinary perspective on professional diagnostic knowledge integrated conceptualizations in medical education and teacher education into an interdisciplinary model with the two dimensions of content-related facets and abstract types of knowledge (Förtsch et al., 2018). The model acknowledges that the issue of content-specificity also affects abstractions like types of professional knowledge, and thus emphasizes limited comparability of professional diagnostic knowledge across disciplines.

We argue nonetheless that interdisciplinary research in diagnosing may still benefit from a more abstracted level of observation, namely: diagnostic practices. We build on the idea of epistemic practices, which are defined as “the specific ways members of a community propose, justify, evaluate, and legitimize knowledge claims within a disciplinary framework” (Kelly, 2008, p. 99). Epistemic practices involve community-specific or discipline-specific epistemic aims (e.g., that a claim is justified), epistemic ideals (standards and criteria to assess the achievement of aims, e.g., that the evidence supports the claim and disconfirms competing claims), and processes that are considered reliable (e.g., disconfirming competing claims; Duncan and Chinn, 2016). Transferring the idea of epistemic practices into the context of diagnosing, we define diagnostic practices as systematic approaches that are applied to collect and integrate case-specific information to reduce uncertainty, and to make and communicate informed and justifiable decisions in a professional situation (Kelly, 2008; Heitzmann et al., 2019). We assume that diagnostic practices within disciplines may involve specificities concerning their epistemic aims, ideals and processes (Duncan and Chinn, 2016), e.g., the standards for justifying a diagnosis. Therefore, comparing diagnostic practices across disciplines may improve our understanding and facilitate future research.

To conceptualize diagnostic practices across different disciplines, we refer to underlying diagnostic activities such as *generating hypotheses*, *generating evidence*, *evaluating evidence*, and *drawing conclusions* (Fischer et al., 2014; Heitzmann et al., 2019; see Supplementary Material section “Supplementary Illustration of the Framework of Diagnostic Activities” for further details). The activities framework has been investigated in different disciplines, e.g., social work education (Ghanem et al., 2018), teacher education (Csanadi et al., 2018), and medical education (Lenzer et al., 2017). We assume, that although concrete hypotheses, evidence, and conclusions are specific, the epistemic purpose of these diagnostic activities is conceptually transferable across disciplines (Hetmanek et al., 2018): Although different hypotheses are appropriate for different diagnostic cases, the activity of generating hypotheses holds the purpose of identifying potential explanations, which may require further investigation. Thus, in investigating diagnostic activities, the case-specific content may be less important compared to characteristics concerning the structure of cases (e.g., the form and amount of potentially available evidence).

As a starting point in investigating diagnostic practices, we can interpret and integrate disciplinary conceptualizations used in previous research in terms of diagnostic activities: In medical education, research has focused in particular on process characteristics of diagnostic reasoning (e.g., Coderre et al., 2003; Norman, 2005; Mamede and Schmidt, 2017). Several studies found that medical students conform to a diagnostic practice, which was characterized as hypothesis-driven approach: Students generated different hypotheses and evaluated evidence accordingly to draw conclusions about their initial hypotheses (e.g., Coderre et al., 2010; Kiesewetter et al., 2013). The hypothesis-driven approach reflects an epistemic ideal of differential diagnosing, which is considered a reliable process in medicine and is thereby taught in medical education (see Duncan and Chinn, 2016). However, research has also found that some medical students exhibit a data-driven approach instead, which focuses on generating and evaluating evidence without considering specific hypotheses or integrating evidence into conclusions (e.g., Gräsel and Mandl, 1993; Norman et al., 2007; Kiesewetter et al., 2013).

In teacher education, research has mostly conceptualized diagnostic practices in terms of professional vision (Goodwin, 1994). Two subcomponents of professional vision have been distinguished: noticing, which includes identifying problems and generating hypotheses, and reasoning, which comprises describing, explaining, and predicting (e.g., Seidel and Stürmer, 2014). *Describing* refers to reporting generated evidence. *Explaining* means evaluating evidence in reference to professional knowledge. Therefore, describing and explaining both focus on evidence and seldom involve generating hypotheses or drawing conclusions, both of which point to *predicting* consequences of observations. Research indicates that expert teachers integrate *describing*, *explaining*, and *predicting* into their diagnostic practice (Seidel and Prenzel, 2007). However, *describing* seems to be a prevailing aspect, while the use of *predicting* is more variant (Stürmer et al., 2016).

Given that work surrounding diagnostic assessment has primarily emerged from the disciplines of medical education and teacher education, we aimed to compare and integrate these two theoretical approaches with respect to diagnostic activities and diagnostic practices. Specifically, we operationalized diagnostic practices as the co-occurrence of diagnostic activities, which we investigated via the use of Epistemic Network Analysis (ENA) (Shaffer, 2017). The research questions are as following:

RQ1: To what extent do learners’ *diagnostic activities* differ between medical education and teacher education?
RQ2: To what extent do learners’ *diagnostic practices* differ between medical education and teacher education?

## Method

### Participants

A total of 142 medical students and 122 pre-service teachers participated in two matched data collections. Medical students were in their 5th to 11th semester (*M* = 8.15; SD = 1.82). Their mean age was *M* = 24.41 (SD = 2.89). A total of 102 were women and 40 were men. Pre-service teachers were in their 1st to 13th semester (*M* = 4.55; SD = 3.40), were on average *M* = 22.96 years old (SD = 4.10), and were mostly women (106 women; 15 men; 1 non-binary). Since half of the sample in teacher education was in their 1st to 4th semester, we defined a subsample of students in teacher education in the 5th or a higher semester for additional subsample analyses (see Supplementary Material section “Supplementary Subsample Analyses”).

### Materials

We developed simulation-based learning environments for medical education and teacher education, using the authoring tool CASUS (Hege et al., 2017). Both learning environments included eight cases with a parallel structure: The cases began with an initial problem concerning a virtual patient or student. Next, learners could freely choose to access several informational sources in any sequence. Learners solved two tasks in each of the eight cases: First, they provided a diagnosis of the virtual patient or virtual student’s problem; second, they had to write a justificatory report, after being prompted, to justify their diagnosis by indicating how they approached and processed the case information.

The medical education cases presented virtual patients with symptoms of fever and back pain. Medical students were asked to take over the role of a general practitioner. After reading the initial problem statement, where the patient revealed his or her reason for seeing a physician, learners accessed the patient’s history and had the option to access the results of different examinations and tests, e.g., physical examination, laboratory, X-ray, ECG.

In the teacher education cases, we asked pre-service teachers to take over the role of a teacher who was encountering a student with some initial performance-related or behavioral problems that might even be clinically relevant, e.g., ADHD or dyslexia. We chose these topics because they are relevant for teachers and at the same time entail structural similarities to medical cases. After reading the initial problem, the learners could access informational sources such as reports of observations from inside and outside of the classroom as well as transcripts of conversations with the student, the parents, and other teachers. Moreover, participants could explore samples of the student’s written exercises and school certificates.

For further details on the learning environment and the cases used, see Supplementary Material sections “Supplementary Case Materials for Medical Education” and “Supplementary Case Materials for Teacher Education.”

### Procedure

The data collection was computer-based and took place in a laboratory setting. We introduced participants to the aims, scope, and procedure of the study and familiarized them with the materials. Next, participants entered the simulation-based learning environment that was designed for their field of study. After giving informed consent to participate in the study, they had to answer a knowledge pretest that took up to 35 min. Afterward, they entered the learning phase, consisting of the eight simulated cases of their respective discipline. Time on task for all cases was *M* = 45.1 min (SD = 12.2) in medical education and *M* = 51.8 min (SD = 16.5) in teacher education. After four cases, participants took a break of 10 min before continuing with the second part of the learning phase and solving cases five to eight. Subsequently, they had to answer a knowledge posttest, which again took up to 35 min. Finally, participants received monetary compensation.

### Data Sources and Instruments

For this paper, we analyzed only the text data from the justificatory reports that all learners wrote for the eight simulated cases. Participants wrote the justificatory reports in an empty text field, right after indicating their diagnosis for each case. There was no template or additional support apart from the standardized prompt to justify the diagnosis by indicating how they approached the case and how they processed the case information. The overall data set used in this paper consisted of 1,136 justificatory reports written by the 142 medical students (average number of words per report *M* = 57.4; SD = 32.6) and 976 justificatory reports written by the 122 pre-service teachers (average number of words per report *M* = 89.6; SD = 53.2).

### Diagnostic Activities

We coded the two sets of justificatory reports on four diagnostic activities: *generating hypotheses*, *generating evidence*, *evaluating evidence*, and *drawing conclusions*. Table 1 presents definitions and examples of the four codes. We developed a coding scheme applicable for medical education and teacher education. Coding and segmentation were done simultaneously to account for overlap in the activities as well. In both disciplines, the raters were first to second year doctoral students and student assistants (minimum 6th semester) from the respective fields. All raters were blind to this study’s research questions. Raters did four rounds of joined coding training, starting with 20 reports and increasing the number in every round of training. To evaluate inter-rater reliability (IRR), five raters in medical education and four in teacher education coded 150 reports for the respective project (13% of the data set in medical education; 15% in teacher education). The overall IRR for the simultaneous segmentation and coding was Krippendorff’s α_U_ = 0.67 in medical education and α_U_ = 0.65 in teacher education (see Table 1), which we consider as satisfactory. For the analyses, we calculated the share of diagnostic activities within medical education and teacher education, respectively, as the percentages of the different diagnostic activities relative to the overall amount.

**TABLE 1:** Definitions, examples, and inter-rater reliabilities (IRRs indicated as Krippendorff’s α_U_) for the four codes: *generating hypotheses*, *generating evidence*, *evaluating evidence*, and *drawing conclusions*.

		Medical education		Teacher education	
Code	Definition	Example	IRR	Example	IRR
Generating hypotheses	Explicit collection of different potential diagnoses or pointing to one diagnosis involving expressed insecurity, e.g., using conjunctive mood.	I believe this is a case of nerve entrapment.	0.60	The initial information makes me think of impaired vision, a reading disorder, or emotional problems as potential explanations for Annika’s issues.	0.43
Generating evidence	Explicit description of accessing informational sources, e.g., tests, interviews, or observations.	Subsequently, I looked at the MRI and X-ray.	0.65	I observed Anna’s school-related behavior and achievement.	0.56
Evaluating evidence	Explicit listing and/or interpretation of separate case information.	Among other results, the patient has an increased CRP and leukocytosis.	0.75	Markus behaves aggressively and gets offended very easily.	0.75
Drawing conclusions	Explicit conclusion or rejection of at least one diagnosis.	The patient clearly has tonsillitis involving a fever.	0.65	Consequently, I rejected the diagnosis of ADHD.	0.49

### Diagnostic Practices

We operationalized diagnostic practices as the co-occurrences of diagnostic activities in the justificatory reports, using the method of ENA (Shaffer, 2017). The ENA algorithm analyzes co-occurring diagnostic activities within a moving window of two sentences (Siebert-Evenstone et al., 2017). Therefore, subsequent to the coding, we determined presence or absence of the four diagnostic activities per sentence. We accumulated the co-occurrences and created one network graph per discipline. In the network graphs, the colored edges refer to co-occurrences between diagnostic activities, with thickness indicating their relative frequencies. Relative frequencies of co-occurring activities allowed us to draw inferences about the general diagnostic practices of each discipline. Additionally, a comparison graph (i.e., showing only the difference between both graphs), allowed us to isolate the differences between the two disciplines’ diagnostic practices.

We also centered the networks and created one centroid per learner as well as per discipline. The centroids’ position is relative to the co-occurrences between diagnostic activities in the respective network. On the level of single learners, the representation of centroids can be used to depict the learners’ distribution within the network space, which can be interpreted as an indicator of interindividual heterogeneity in diagnostic practices. On the level of disciplines, we can consider centroids as group means. ENA enables statistical testing of the group differences in overall diagnostic practices between learners in medical education and teacher education. To facilitate the testing of the group differences, we used the option of means rotation, which aligns the two disciplines’ group means on the X-axis, thus depicting systematic variance on only one dimension.

### Statistical Analyses

To address RQ1, the extent to which diagnostic activities differ between learners from medical education and teacher education, we calculated t tests for independent samples, one test per diagnostic activity, using Bonferroni-adjusted alpha levels of α = 0.0125 per test (α = 0.05/4). To statistically test RQ2, differences in diagnostic practices between learners from medical and teacher education, we used an independent-samples t test as well, comparing the two group means from the two disciplines’ ENA networks at an alpha level of α = 0.05. If Levene’s test indicated unequal variances, we adjusted the degrees of freedom accordingly.

## Results

Comparing the two disciplines, there was a significant difference regarding the number of semesters studied (medical education *M* = 8.15; SD = 1.82; teacher education *M* = 4.55; SD = 3.40), *t*(173) = 10.35, *p* < 0.001, Cohen’s *d* = 2.75. Therefore, we analyzed the relation with the percentages of diagnostic activities within the disciplines. There was no significant correlation found between number of semesters studied and the percentages of the different diagnostic activities (for details see Supplementary Material section “Supplementary Results of a Correlation Between Semesters Studied and Number of Diagnostic Activities”). However, to ensure that the number of semesters studied did not bias the results, we performed the following analyses not only with the full sample as reported in the following sections, but a second time, comparing learners from medical education to the specified subsample of learners from teacher education in their 5th or a higher semester (see Supplementary Material section “Supplementary Subsample Analyses”).

### Diagnostic Activities in Medical Education and Teacher Education (RQ1)

In both disciplines, *evaluating evidence* was clearly the most prominent activity found in the justificatory reports with a share of more than half of the diagnostic activities found in the reports (medical education *M* = 60.96%; SD = 10.24%; teacher education *M* = 66.08%; SD = 17.02%). The difference in the relative frequencies for *evaluating evidence* was significant with a small effect size [*t*(192) = 2.91, *p* = 0.004, Cohen’s *d* = 0.37]. We found that in medical education, the share for *generating hypotheses* was about twice as high (*M* = 16.26%; SD = 7.96%) as in teacher education (*M* = 8.37%; SD = 6.41%). This difference was significant with a large effect size [*t*(261) = 8.92, *p* < 0.001, Cohen’s *d* = 1.08]. By contrast, the share for *generating evidence* was about twice as high in teacher education (*M* = 13.74%; SD = 14.81%) as in medical education (*M* = 6.79%; SD = 8.26%), and this was also significantly different with a medium-sized effect [*t*(183) = 4.60, *p* < 0.001, Cohen’s *d* = 0.59]. In medical education, we also found a significantly higher share for *drawing conclusions* (*M* = 15.99%; SD = 6.39%) than in teacher education (*M* = 11.82%; SD = 6.83%), with a medium effect size [*t*(262) = 5.13, *p* < 0.001, Cohen’s *d* = 0.63].

Comparing medical education with the specified subsample from teacher education (see section “Participants”), the results show the same results pattern (for detailed results see Supplementary Material section “Supplementary Subsample Analyses”). However, there was no significant difference in the relative frequencies for *evaluating evidence* [medical education *M* = 60.96%; SD = 10.24%; teacher education *M* = 65.40%; SD = 18.00%; *t*(77) = 1.81, *p* = 0.075, Cohen’s *d* = 0.34].

### Diagnostic Practices in Medical Education and Teacher Education (RQ2)

**FIGURE 1:**
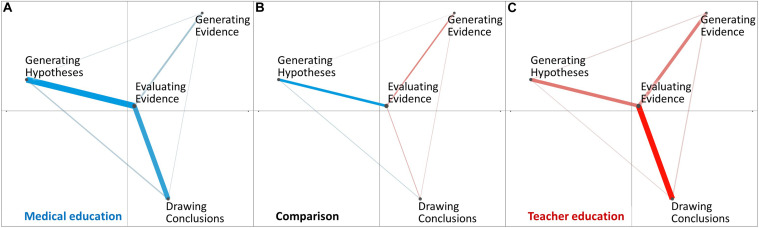
ENA networks from medical education **(A)**, and teacher education **(C)**. The comparison network **(B)** depicts only the differences between the other two networks.

In Figure 1, we present the diagnostic practices of learners from medical education (Figure 1A) and teacher education (Figure 1C) as network graphs. The colored edges and their thickness reflect the relative frequencies of co-occurrences of diagnostic activities. The overall network across all learners from medical education (Figure 1A) showed some similarities to the overall network across all learners from teacher education (Figure 1C): First, in both disciplines, we found that the relative frequencies of co-occurrences were in accordance with the relative frequencies of the individual diagnostic activities (see the results for RQ1). In both network graphs, the three relatively most frequent co-occurrences were the ones including *evaluating evidence*. This is why we found *evaluating evidence* near the center of the disciplines’ overall networks. However, by looking at its temporal context indicated by co-occurrences with other diagnostic activities, we can draw inferences about the purpose of *evaluating evidence* within the respective context. When it co-occurs with *drawing conclusions* or *generating hypotheses*, *evaluating evidence* serves the purpose of *explaining*; whereas when co-occurring with *generating evidence*, *evaluating evidence* may rather *describe* the evidence (see Table 2 for examples). To compare learners from medical education and teacher education, the comparison graph (Figure 1B) shows the difference between the two disciplines’ overall networks, therefore indicating only the differences in co-occurrences. In medical education, there was a relatively higher frequency of *evaluating evidence* co-occurring with *generating hypotheses*, pointing to a rather hypothesis-driven approach that puts more emphasis on *explaining* evidence; whereas learners in teacher education exhibited a relatively higher frequency of co-occurrences between *evaluating evidence* and *generating evidence*, indicating a tendency toward *describing* evidence or a data-driven approach.

**TABLE 2:** Examples of *evaluating evidence*, co-occurring with *generating evidence*, *generating hypotheses*, or *drawing conclusions* in a temporal context of one to two sentences in the disciplines of medical education and teacher education.

Case	Text	Generating hypotheses	Generating evidence	Evaluative evidence	Drawing conclusions
**Section a: Examples of *evaluating evidence* co-occurring with *drawing conclusions* or *generating hypotheses* in the discipline of medical education**					
2	Due to his age and the sudden symptomatology in only his lumbar spine, I would diagnose a rheumatic disease.	0	0	1	1
7	Upon physical examination, she mostly indicated pain in the upper abdomen, which highlights the region of the liver, gall bladder, and eventually the biliary tract and pancreatic duct.	0	0	1	0
	Laboratory results indicated increased liver values, which is why I believe the patient has hepatitis.	1	0	1	0
**Section b: Examples of *evaluating evidence* co-occurring with *drawing conclusions* or *generating hypotheses* in the discipline of teacher education**					
8	The characteristic writing, confusion of characters, deficits in stringing together syllables, as well as deficits in syllabification and slow reading speed, combined with an otherwise good school performance, clearly indicate dyslexia.	0	0	1	1
6	Thomas might have eventually developed ADHD and therefore low concentration.	1	0	0	0
	This assumption is backed by the fact that his performance in all subjects decreased and that he does not fully answer all questions on exams.	0	0	1	0
**Section c: Examples of *evaluating evidence* co-occurring with *generating evidence* in the discipline of medical education**					
7	First, I examined all the available information, before focusing on the most relevant points.	0	1	0	0
	They mostly seemed to be related to the liver.	0	0	1	0
8	Even after being treated by the general practitioner, the patient still had a fever and symptoms of a systemic infection.	0	0	1	0
	This is why, considering the anamnesis regarding previous travels, I decided to administer an HIV test.	0	1	1	0
**Section d: Examples of *evaluating evidence* co-occurring with *generating evidence* in the discipline of teacher education**					
6	I examined the teacher’s report and the available documents.	0	1	0	0
	It seems that Thomas’ symptoms have only been observable recently and that he has repeatedly complained about small font sizes.	0	0	1	0
5	Initially, I collected information from observations, conversations, the annual report, and recent school exams.	0	1	0	0
2	My attention was caught by the mother’s description of her reading behavior at home, especially in terms of reading aloud.	0	0	1	0

**FIGURE 2:**
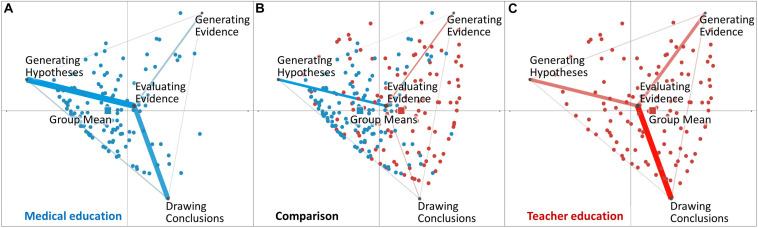
Distributions of learners within medical education **(A)**, and teacher education **(C)**. The figures also contain group means (squares) across the learners within the two disciplines. The comparison graph **(B)** depicts both distributions and the differences between the other two networks.

In addition to the disciplines’ overall networks, Figure 2 presents the distribution of single learners across the two disciplines’ overall networks. The colored points represent the networks’ centroids on the level of single learners from medical education (Figure 2A) and teacher education (Figure 2C). In teacher education, single learners’ centroids (red colored points) are more scattered across the network space, compared to the positioning of the single learners’ centroids in medical education (blue colored points). This indicates that the diagnostic practices of learners from medical education are more homogeneous compared with the diagnostic practices of learners from teacher education.

Figure 2 presents centroids on the group level, representing the means of all learners within the two disciplines of medical education and teacher education as indicated by the colored squares. The positioning of the group mean of learners from medical education (*M* = −0.36, SD = 0.63, *N* = 142) was statistically significantly different from the positioning of the group mean of learners from teacher education [*M* = 0.42, SD = 0.74, *N* = 122; *t*(240.48) = −9.16, *p* < 0.01, Cohen’s *d* = 1.14]. This result indicates a significant difference in diagnostic practices between teacher education and medical education. Repeating these analyses, comparing students from medical education with the specified subsample from teacher education, revealed basically the same result (for details see Supplementary Material section “Supplementary Subsample Analyses”).

## Discussion

In analyzing learners’ reports of their diagnostic activities in medical education and teacher education, we found that future physicians and future teachers put the most focus toward *evaluating evidence*. Moreover, learners from teacher education focused more on *generating evidence*, whereas learners from medical education put more focus toward *generating hypotheses* and *drawing conclusions*. These results support the notion that the relative emphasis on each diagnostic activity differs between these disciplines.

The disciplinary differences in the use of diagnostic activities is also reflected by overall diagnostic practices. Because the overall network across all learners from medical education was similar to the network across all learners from teacher education, this similarity suggests that the overall diagnostic practices are similar. Still, there were significant disciplinary differences in the relative frequencies of the co-occurrences of diagnostic activities. In general, we found that learners from medical education showed a more explanation-driven or hypothesis-driven approach (see Coderre et al., 2010; Kiesewetter et al., 2013; Seidel and Stürmer, 2014), whereas learners from teacher education showed a more description-driven or data-driven approach (see Gräsel and Mandl, 1993; Norman et al., 2007; Kiesewetter et al., 2013; Seidel and Stürmer, 2014). Furthermore, learners from teacher education showed greater variability in their diagnostic practices than learners from medical education.

We interpret the results relating to epistemic ideals as the “criteria or standards used to evaluate epistemic products” (Duncan and Chinn, 2016, p. 158). In the context of medical education, differential diagnosing is considered as ideal for ensuring a reliable process. Differential diagnosing essentially refers to a hypothesis-driven approach of generating and testing hypotheses (see Fischer et al., 2014), which is what we observed in learners from medical education. This diagnostic standard is put into practice on different levels (e.g., in guidelines and university curricula), and is systematically taught to future physicians in their medical programs. In teacher education, we are not aware of a widespread use of such specific standards for diagnosing in general and particularly regarding the topic of students’ behavioral and performance-related disorders. Research in teacher education was referred to as a rather “young” field (Grossman and McDonald, 2008) and thus, the evolvement of standards for diagnosing might be less advanced than in medical education. In comparison with medical students, pre-service teachers also seem to show greater variability in their diagnostic practices, which may support the notion of lower standardization in diagnostic practices or at least in educating pre-service teachers to apply diagnostic practices. However, there might be some implicit ideals that enhance pre-service teachers’ tendency to embrace a data-driven approach in their diagnostic practices. First, as a reaction to findings of teachers’ biases in diagnostic tasks (e.g., Südkamp et al., 2012), some teacher education programs have subsequently taught the concept of professional vision (Goodwin, 1994) to pre-service teachers, emphasizing the need to focus on *describing* observations before *explaining* them (e.g., Seidel and Stürmer, 2014). This development may complement other implicit values (see Duncan and Chinn, 2016) in teaching, such as to avoid being judgmental toward students (Aalberts et al., 2012). Therefore, the findings may reflect disciplinary differences in epistemic ideals implemented in higher education and diagnostic practices, respectively.

### Limitations

One limitation of the study involves the inter-rater reliabilities for *generating hypotheses* and *drawing conclusions*, which were relatively low in the teacher education data. This could limit the conclusions that can be drawn about the variability in diagnostic practices of teacher education learners in particular.

Another limitation may be the learners’ study progress: In the full sample, learners from medical education had completed significantly more semesters than learners from teacher education. However, the number of semesters did not correlate with the proportion of the different diagnostic activities. The subsample analyses, which compared students from medical education with students from teacher education in their 5th or a higher semester revealed the same patterns of results as the analyses of the full sample. Hence, it seems unlikely that the *a priori* difference in the number of semesters would lead to substantial bias in our results.

Furthermore, we acknowledge that although we argue for the interdisciplinary comparability of the diagnostic activities’ epistemic purpose, this conceptualization may still not fully eliminate the issues associated with comparing disciplinary diagnostic practices. Yet, we think that diagnostic activities and diagnostic practices are more advantageous in terms of interdisciplinary comparability than other investigated approaches, e.g., professional diagnostic knowledge.

The choice of clinical topics in both disciplines served the purpose of having similarly structured problems. Nevertheless, in teacher education there are other than clinical areas where diagnosing is relevant (e.g., assessing a student’s level of skill). Thus, our choice might limit the generalizability of the findings to other areas of assessment in teacher education. However, if we consider diagnostic practices as discipline-specific approaches, it is reasonable to assume that the findings may replicate in other areas of teachers’ diagnostic assessments, which could be investigated in further research.

Finally, similar to verbal protocols, assessing reported activities raises the question of validity, concerning the degree to which the reports effectively represent actually performed activities. Therefore, further research might additionally complement reported diagnostic activities with behavioral data like user-logs.

## Conclusion

In this article, we have argued that interdisciplinary research on diagnostic assessments benefits from comparisons drawn at the level of diagnostic activities (Fischer et al., 2014) and diagnostic practices (Kelly, 2008; Heitzmann et al., 2019) as comparing professional diagnostic knowledge has been found to be difficult due to its content specificity. In an interdisciplinary comparison of justifications by learners from teacher education and medical education, we found significant differences in their diagnostic activities and diagnostic practices. We found a more hypothesis-driven approach in justifications of learners from medical education, who put relatively more emphasis on generating hypotheses and drawing conclusions. Learners from teacher education instead seemed to apply a more data-driven approach, with a stronger focus on generating and evaluating evidence. The results may allude to different epistemic ideals and diagnostic standards (see Duncan and Chinn, 2016) taught in higher education and thereby put into diagnostic practices.

Diagnostic activities can provide a useful and interdisciplinary framework to analyze diagnostic practices across disciplines. For future interdisciplinary research, we recommend considering matched study designs, as implemented in our project, to maximize interdisciplinary comparability. Additionally, from a practically oriented viewpoint, we recommend that educators from both the medical education and teacher education fields reflect further on their standards in diagnosing and their underlying epistemic ideals to further increase the awareness of practitioners and systematization in teaching. Finally, we encourage researchers to further investigate the potential relation between epistemic ideals and diagnostic practices in terms of interdisciplinary differences, commonalities, and their continuing evolvement.

## Data Availability Statement

The raw data supporting the conclusions of this article will be made available by the authors. Requests to access the data should be directed to elisabeth.bauer@psy.lmu.de.

## Ethics Statement

The studies involving human participants were reviewed and approved by the Ethics Committee of the Medical Faculty of LMU Munich (no. 17-249). The participants provided their written informed consent to participate in this study.

## Author Contributions

EB, FF, MF, JK, MS, and JZ developed the study concept and contributed to the study design. EB and MS performed the data analysis. EB, FF, and MS interpreted the data. EB drafted the manuscript. FF, MF, JK, MS, DS, and JZ provided critical revisions. All authors approved the final version of the manuscript for submission.

## Conflict of Interest

The authors declare that the research was conducted in the absence of any commercial or financial relationships that could be construed as a potential conflict of interest.
