# Supplementary material for: Diagnostic Activities and Diagnostic Practices in Medical Education and Teacher Education: An Interdisciplinary Comparison
Source: Front Psychol. 2020 Oct 20;11:562665. doi: 10.3389/fpsyg.2020.562665 (PMC7606905; doi:10.3389/fpsyg.2020.562665)
Supplement: Supplementary file 1 [file Data_Sheet_1.PDF]

Supplementary Material

1 Supplementary illustration of the framework of diagnostic activities

In our research, we refer to a framework of diagnostic activities such as generating hypotheses, generating evidence, evaluating evidence, and drawing conclusions (Fischer et al., 2014; Heitzmann et al., 2019). The full framework includes eight diagnostic activities, which are illustrated in the supplementary Table 1.

Supplementary Table 1. The framework of diagnostic activities, adapted from Heitzmann et al., 2019.

**Diagnostic Activity**	**Examples from medical education and teacher education**
Problem identification	A physician encounters a patient who reports non-specific symptoms such as shortness of breath; A teacher faces a student who wrongly answers a question in class.
Questioning	A physician asks what the reason for the symptoms could be; A teacher asks what the reason for a student’s error could be.
Hypothesis generation	A physician suspects a specific disease, such as a pulmonary embolism; A teacher suspects a specific misconception.
Construction and redesign of artefacts	A medical report which indicates the need for further examination, e.g. a computer tomography; The development of a task which provides insight into the presence of a misconception.
Evidence generation	Conducting further examination, for example through computed tomography; Observation of the student’s solution of the task.
Evidence evaluation	Evaluation of the computer tomography with signs of a pulmonary embolism; Evaluation of the solution of the task with some but not all of the signs for the hypothesized misconception.
Drawing conclusions	Deciding that the most likely cause of the patient’s symptoms is a pulmonary embolism; Deciding that the most likely reason for the student’s error is the assumed misconception, which impedes further learning.
Communication and scrutinization	A medical report with the diagnosis of a pulmonary embolism for another physician; Informing another teacher about the discovered misconception held by a certain student so that teacher can adapt the teaching.

2 Supplementary case materials for medical education

The medical education cases presented virtual patients with symptoms of fever and back pain and medical students were asked to take over the role of a general practitioner. One exemplary case was about a 36 year-old female, Mrs. Hoffmann, who had a febrile and flu-like infection for almost a week before seeing the doctor. In addition, she experienced fatigue, loss of appetite, sickness and diarrhea. One month earlier, she returned from a trip to Costa Rica, for which she received the recommended vaccinations prior to departure. The anamnesis provided the information that no other persons in her surrounding had the same symptoms; that she does not know about any pre-existing illnesses and does not consume any prescribed drugs, apart from occasionally using homeopathic globules; and that, she is allergic to penicillin and nickel; moreover, she is a non-smoker, occasionally consumes alcohol, and excluded the option of pregnancy. To gather more information, learners could access the patient's history and had the option to access different tests and test results, e.g. physical examination, laboratory, x-ray, ECG, HIV test, and others (see supplementary Figure 1). Overall, the symptoms and test results point to an acute hepatitis A infection. Typical symptoms of the patient's current stage of the infection are fatigue, limb pain, fever, sickness, diarrhea and joint pain.

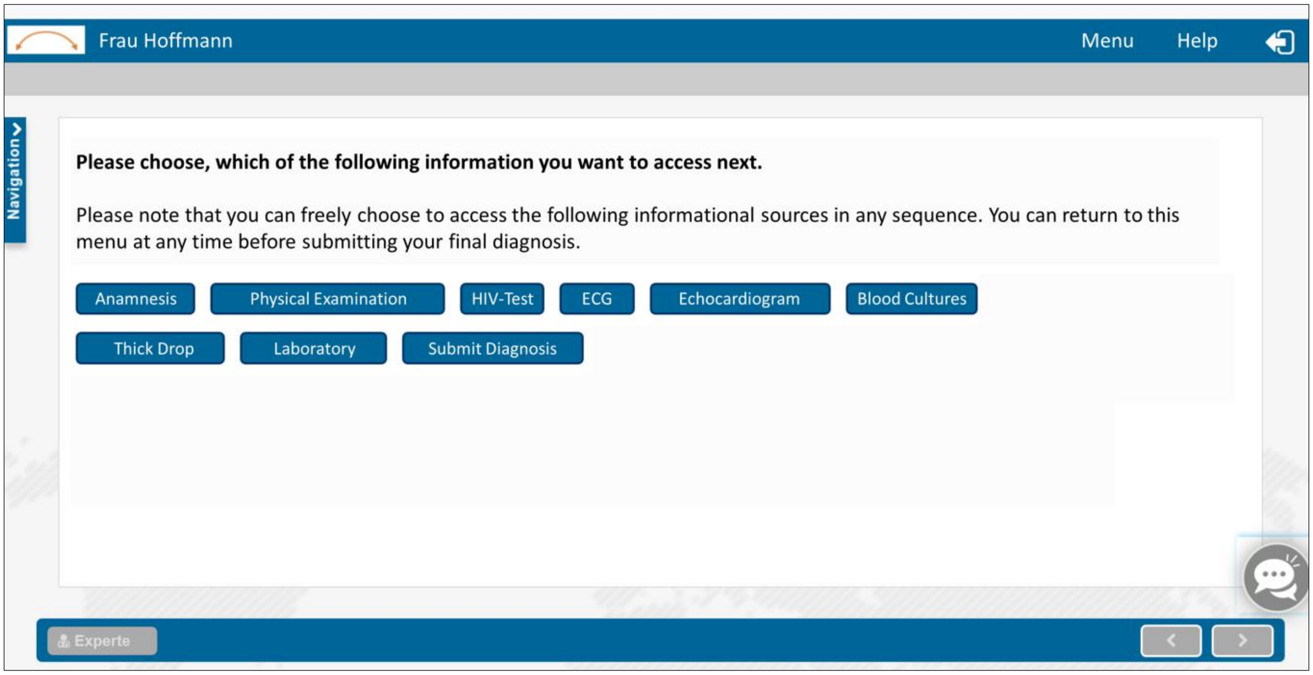

Supplementary Figure 1. Screenshot of user interface for the medical education case in the CASUS learning environment.

3 Supplementary case materials for teacher education

In the teacher education cases, we asked pre-service teachers to imagine themselves in the position of a teacher who was encountering a student with some initial performance-related or behavioral problems that might even be clinically relevant, e.g. ADHD or dyslexia. One example is the case of a secondary student named Anna who is displaying symptoms of an attention-deficit disorder. The leaners are asked to put themselves into the role of Anna's class teacher, who teaches German classes and music lessons. The initial problem statement for the case describes Anna as a 5th grade student,

eleven years old, who constantly needs to be pushed to finish her tasks and who has bad grades in many subjects, especially the main subjects. The learners could examine written observations of Anna's in-class and out-of-class behavior, read recordings of conversations with Anna, or with her parents and several teachers, or look at Anna's last annual report and an example of a written exercise (see supplementary Figure 2). Her behavior is described as very calm and distracted. She is slow in reading and it is difficult for her to answer questions about a text that she just read. She often fails to fulfill the exact instruction of a task or fails to fully complete a task. Moreover, she often does not bring all required school supplies or comes late in the mornings. In a parent-teacher meeting, Anna's mother backs up the impression of a disorganized and slow learning behavior when talking about the homework situation. Anna's last annual report and the conversations with the other teachers show that her grades are mostly affected by her inattentiveness as well, with the exception of artistic subjects and gym classes. Anna mostly interacts with her one friend and is rather distanced from the other students. Anna herself points out that it is hard for her to concentrate since she feels easily distracted. However, at home, where there are fewer ambient noises, she can focus on and enjoy reading, drawing and painting. Overall, the case information was designed in a way that, the diagnosis of an attention-deficit disorder is the most likely clinical diagnosis, despite several differential diagnoses being relevant.

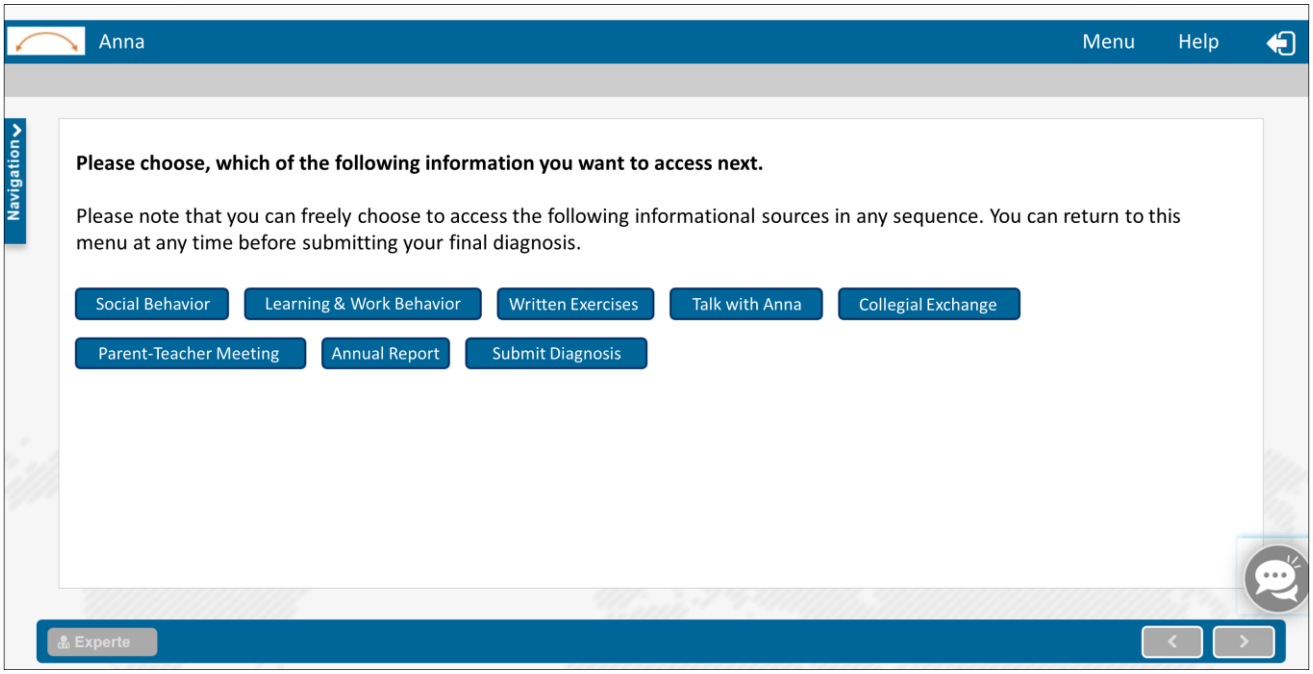

Supplementary Figure 2. Screenshot of user interface for the teacher education case in the CASUS learning environment.

4 Supplementary results of a correlation between semesters studied and number of diagnostic activities.

We analyzed the relation between the relative percentages of diagnostic activities within the disciplines and number of semesters completed. There was no significant correlation found between number of semesters studied and the percentages of the different diagnostic activities. The correlation coefficients and p-values are presented in the supplementary Table 2.

Supplementary Table 2. Results of the two Pearson correlation analyses with the variables *semester* and *percentages of diagnostic activities* within the disciplines of medical education (section a) and teacher education (section b).

		Generating hypotheses	Generating evidence	Evaluating evidence	Drawing conclusions
Section a: Medical Education (*N* = 142)					
Semester	Pearson’s r	.009	-.129	.105	-.012
	p-value	.917	.127	.215	.885
Section b: Teacher Education (*N* = 119)					
Semester	Pearson’s r	.014	.104	-.034	-.140
	p-value	.876	.262	.716	.128

5 Supplementary subsample analyses

Since half of the sample of pre-service teachers were in their 1st to 4th semester, which was excluded in medical education, we defined a comparative subsample of 61 pre-service teachers in their 5^{ht} to 13th semester (*M* = 7.38; *SD* = 2.30), who were on average *M* = 24.20 years old (*SD* = 3.55), and were mostly women (52 women; 8 men; 1 nonbinary). The comparative subsample of 61 pre-service teachers in the 5th or a higher semester accounted for 488 justificatory reports (average number of words per report *M* = 94.3; *SD* = 62.3).

5.1 Diagnostic activities in medical education and teacher education (RQ1)

Comparing medical education with the subsample of 5th or higher semester students from teacher education, there was no significant difference in the relative frequencies for *evaluating evidence* (medical education *M* = 60.96%; *SD* = 10.24%; teacher education *M* = 65.40%; *SD* = 18.00%; *t*(77) = 1.81, *p* = .075, Cohen's *d* = 0.34). Concerning the other three diagnostic activities, the differences between the disciplines was significant: In medical education, the share for *generating hypotheses* was still about twice as high (*M* = 16.26%; *SD* = 7.96%) as in teacher education (*M* = 8.50%; *SD* = 5.50%), with a significant, large-sized effect (*t*(161) = 8.00, *p* < .001, Cohen's *d* = 1.06). The share for *generating evidence* was still about twice as high in teacher education (*M* = 15.00%; *SD* = 15.80%) as in medical education (*M* = 6.79%; *SD* = 8.26%), with a significant medium-sized effect (*t*(74) = 3.84, *p* < .001, Cohen's *d* = -0.74). In medical education, we still found a significantly higher share for *drawing conclusions* (*M* = 15.99%; *SD* = 6.39%) than in teacher education (*M* = 11.10%; *SD* = 7.15%), with a medium effect size (*t*(201) = 4.82, *p* < .001, Cohen's *d* = 0.74).

5.2 Diagnostic practices in medical education and teacher education (RQ2)

In the supplementary Figure 3A and 3B, we compared students from medical education with the subsample of 5th or higher semester students from teacher education. The positioning of the group mean of learners from medical education (*M* = -.21, *SD* = .60, *N* = 142) was statistically significantly different from the positioning of the group mean of learners from teacher education in their 5th or a higher semester (*M* = .49, *SD* = .68, *N* = 61; *t*(100.89) = 6.97, *p* < .01, Cohen's *d* = 1.13).

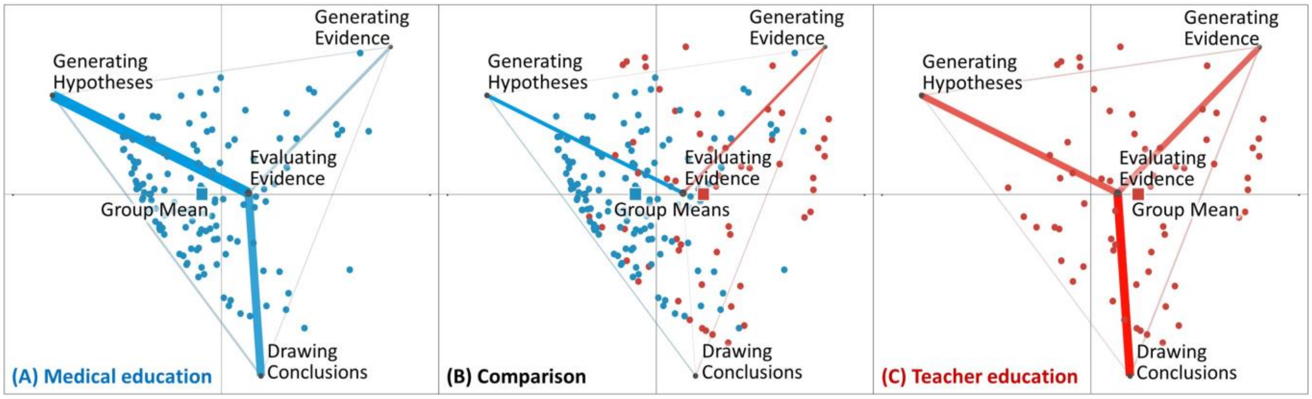

Supplementary Figure 3. ENA networks and distributions of learners from medical education **(A),** and teacher education **(C)**. The figures also contain group means (squares) across the learners within the two disciplines. The comparison graph **(B)** depicts both distributions and the differences between the other two networks.

The results of this subsample analysis support the previously described findings. Furthermore, in the supplementary Figure 3C, we additionally distinguished between students from teacher education who are in their 1st to 4th semester and students from teacher education who are in their 5th or a higher semester. As indicated by the positioning of the two group means displayed in the supplementary Figure 3C, the two subsamples of students from teacher education are overall rather similar.

This re-analysis, which compared students from medical education with a subsample of students from teacher education in their 5th or a higher semester of study, supported the findings from the initial analyses of the full sample, apart from the significant difference in evaluating evidence.
